# Why does Japan have so few cases of COVID‐19?

**DOI:** 10.15252/emmm.202012481

**Published:** 2020-04-28

**Authors:** Akiko Iwasaki, Nathan D Grubaugh

**Affiliations:** ^1^ Department of Immunobiology Yale University School of Medicine New Haven CT USA; ^2^ Howard Hughes Medical Institute Chevy Chase MD USA; ^3^ Department of Epidemiology of Microbial Diseases Yale School of Public Health New Haven CT USA

**Keywords:** Immunology, Microbiology, Virology & Host Pathogen Interaction

## Abstract

The COVID‐19 pandemic has spread to many countries around the world, but the infection and death rates vary widely. One country that appeared to have kept the infection under control despite limited societal restrictions is Japan. This commentary explores why Japan may have, up to now, been spared an escalation of the SARS‐CoV‐2 infections.

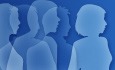

**Despite early exposure, its dense and aging population, and little social distancing measures, Japan reports low infection and low death from COVID‐19. Here, we speculate on and discuss the possible reasons that may account for this anomaly.**

There is a lot of interest brewing as to why Japan has such low numbers of confirmed infected cases of the COVID‐19 disease, caused by the SARS‐CoV‐2 virus (Fig 1), despite its high population density (over 6,100 persons/sqkm in Tokyo, 2.4 times higher than New York City) and large percentage of high‐risk individuals over 65 years of age (about 26%, compared with 15% in the USA). In Singapore and Hong Kong, rapid and strict quarantine rules and contact tracing have helped to “flatten the curve”. In South Korea, mass testing and quarantine measures appear to have reduced the rate of new cases. However, Japan has not engaged in expansive testing, contact tracing, or strict quarantine measures and yet is reporting a slow growth rate of infected persons and a death rate that is currently just 1/10^th^ of world average. It is difficult to make direct comparison of infection rates, because the number of tests per capita varies dramatically between countries. However, this low death rate cannot be simply explained by lack of testing or reporting, as no surge in death from respiratory syndromes has been reported either.

**Figure 1:**
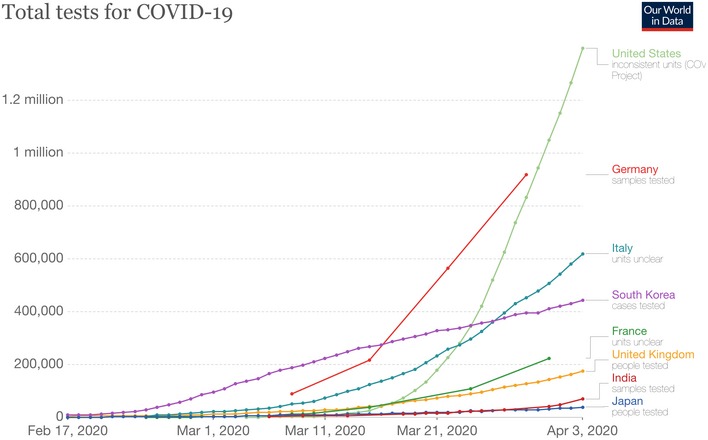
Cumulative number of cases, by number of days since February 1, 2020 Source: https://ourworldindata.org/coronavirus#confirmed-covid-19-deaths-by-country, accessed 6‐4‐2020, data based on European Centre for Disease Prevention and Control.

So how has Japan dealt with COVID‐19? The Japanese Cluster Response Team of the Ministry of Health set forth on March 9^th^ a three‐pronged approach.

• early detection of and early response to infection clusters
• early patient diagnosis and enhancement of intensive care and the securing of a medical service system for the severely ill
• behavior modification of citizens (including advise to refrain from holding large‐scale events, temporary school closures)

Note that none of these involve strict social distancing measures taken by other countries. Why is this? What can we learn from Japan to help flatten the curve in other countries?

Here, we discuss several hypotheses and provide arguments for or against each hypothesis.

1 Japanese culture is inherently suited for social distancing, and face mask use prevents viral spread.

It is certainly true that the Japanese customs do not involve handshaking, hugging, or kissing when greeting. In addition, many Japanese wear cloth or paper face masks (not the N95 respirators required for exclusion of aerosol viral particles) in the winter to avoid transmission of respiratory infections. People use the mask to avoid spreading the infection and also in an attempt to prevent exposure to infection. However, we are unconvinced that this is the main or only reason why COVID‐19 is so well contained in Japan. There is no social distancing in rush hour trains and buses, or when walking in crowded streets to school or to work. The use of face mask is also practiced in other Asian countries that witnessed higher rates of infection. A hint to whether this is a valid hypothesis comes from looking at other pandemic viral respiratory diseases. The community *R*_0_ rate for the 2009 pandemic flu for Japan was 1.28 while USA was 1.7–2.0 (Boelle *et al*, 2011). Thus, *R*_0_ in Japan was somewhat lower than the global median *R*_0_ of 1.47. In addition, an observational study of elementary school children in Japan found that wearing masks had significant protective association (odds ratio of 0.859, 95% confidence interval 0.778–0.949) against seasonal influenza (Uchida *et al*, 2017). Therefore, the social practice culture of Japan and mask use may explain to some extent the lower number of observed COVID‐19 cases, but is unlikely the only explanation.

2 Japanese people were exposed to a milder version of SARS‐CoV‐2 that conferred herd immunity before the spread of a more virulent strain of CoV2.

While possible, there is no current evidence that milder strains of SARS‐CoV‐2 exist. Nor do we know what sort of antibody response would develop as a result of exposure to such a hypothetical variant. Phylogenetic analysis of SARS‐CoV‐2 of more than 3,500 SARS‐CoV‐2 genomes from around the world, including 29 from Japan, suggests that the outbreak in Japan was sparked by several independent virus introductions primarily from China (https://nextstrain.org/ncov?f_country=Japan, accessed 7.4.2020) (Hadfield *et al*, 2018). Furthermore, all of the SARS‐CoV‐2 genomes are highly similar; most contain no more than 10 mutations compared to the virus that started the original outbreak. Thus, it is highly unlikely that the virus has evolved a significantly different phenotype, and even less likely that it was introduced early into Japan.

Notably, early cases in Japan (January–February) were all linked to virus introduced from China. Now, in March, the outbreaks in Japan are linked to introductions from Europe, and there is a large gap in between those early introductions in January and the recent ones in March. While the data are still limited, this suggests that Japan was able to control the early outbreaks, keeping cases down, but is now experiencing a second wave introduced more recently from Europe.

3 Japanese people have reduced susceptibility due to ACE2 receptor expression.

SARS‐CoV‐2 utilizes ACE2 as a receptor to enter cells. It is theoretically possible that ACE2 expression in the respiratory tract is somehow lower in the Japanese population, though no direct evidence was identified during studies investigating coronavirus S‐protein binding‐resistant ACE2 mutants among different populations. If anything, East Asian populations were reported to have higher allele frequency in the ACE2 variants associated with higher ACE2 expression in tissues (Cao *et al*, 2020). However, the only way to find out whether the expression of ACE2 is indeed different is through surface protein staining of lung tissues, which has yet to be done.

4 Japanese people have distinct HLA that confers immune resistance to CoV2.

Genome‐wide association studies (GWAS) conducted on disease susceptibility show that HLA is usually the top locus  associated with disease. This is true for infectious diseases, autoimmunity, or neurological disorders. HLA stands for human leukocyte antigen and is also known as MHC, or major histocompatibility complex. These genes encode for proteins that present antigenic peptides to T cells. HLA class I presents antigenic peptides to CD8 T cells, while HLA class II presents peptides to CD4 T cells. HLA genes are the most highly polymorphic genes in the human genome. The variety in HLA enables our immune system to survey for maximal number of antigen peptides that are present in pathogens, so as to elicit robust cellular immune responses. Previous studies have identified HLA‐B*4601 to be associated with higher risk of developing SARS disease (Lin *et al*, 2003), based on a small number of cases. However, whether there are any HLA alleles that confer resistance to COVID‐19 and whether the allele frequency is higher in the Japanese population are unknown.

5 BCG vaccine used in Japan confers protection against COVID‐19.

Japan, like many other countries including China, Korea, India, and the Russian Federation, have mandatory childhood BCG vaccines against tuberculosis. These countries have so far a relatively low per capita death rate from COVID‐19 compared to countries that have no mandatory BCG vaccines (USA, Spain, France, Italy, The Netherlands). What further distinguishes Japan is that the BCG vaccine strain used in Japan, Brazil, and Russia is one of the original strains, while further modified BCG strains are used for vaccination in European countries. This association between BCG vaccination and apparent low COVID‐19 incidence in Japan has spurred the idea that these two things may be linked (for more discussions on this topic, visit https://www.jsatonotes.com/2020/03/if-i-were-north-americaneuropeanaustral.html and https://news.yahoo.co.jp/byline/kimuramasato/20200405-00171556/).

How would BCG, an attenuated bacterial vaccine completely unrelated to COVID‐19, provide protection? Michai Netea and colleagues hypothesized that the vaccine may boost “trained immunity” (Netea *et al*, 2016)—in other words, certain immune stimuli may induce a prolonged state of resistance against pathogens in general, by elevating the expression levels of resistance factors. Studies have shown that receipt of BCG vaccine was associated with a reduction in all‐cause mortality within the first 1–60 months: The average relative risks were 0.70 (95% confidence interval 0.49–1.01) from five clinical trials (Higgins *et al*, 2016). Furthermore, Netea and colleagues showed that BCG vaccination reduced the levels of viremia caused by the yellow fever virus live attenuated vaccine (Arts *et al*, 2018), and post‐BCG increase of IL‐1β production strongly correlated with lower viremia after yellow fever virus administration. A placebo‐controlled randomized clinical trial of 1,000 healthcare workers in The Netherlands has started, and a similar trial is planned to begin at the Max Planck Institute (de Vrieze, 2020). The outcomes of these trials will help us to understand whether and how BCG confers resistance to other pathogens including SARS‐CoV‐2.

## Conclusion

There are many other theories to explain the low number of COVID‐19 cases in Japan, yet we still do not have enough information to determine the cause of this striking discrepancy. Clearly, we do not understand what causes these differences. Many of these hypotheses can be tested as suggested above, such as examining ACE2 expression levels in the respiratory tract, GWAS data on COVID‐19 susceptibility, and whether BCG vaccines indeed confer long‐term innate immune resistance to SARS‐CoV‐2. The three‐pronged approach by the Cluster Response Team of the Japanese Ministry of Health has thus far contained the spread of COVID‐19 by quickly identifying clusters of infections, testing, and quarantine of the infected individuals. A word of caution is whether this approach will work in cases where super‐spreaders ignite a large‐scale transmission, or when there are multiple clusters that occur throughout the country at once. Perhaps one of the reasons for the low number of cases in Japan might relate to lack of super‐spreader events to date. Just within the last 24 h, Japan has declared the state of emergency, as Tokyo faces more than 1,000 confirmed cases, more than double the number a week ago. Perhaps stronger social distancing measures are required to keep the curve flattened in Japan.
